# Prospective evaluation of GeneXpert for the diagnosis of HIV- negative pediatric TB cases

**DOI:** 10.1186/s12879-015-0814-2

**Published:** 2015-02-18

**Authors:** Do Chau Giang, Tran Ngoc Duong, Dang Thi Minh Ha, Ho Thi Nhan, Marcel Wolbers, Nguyen Thi Quynh Nhu, Dorothee Heemskerk, Nguyen Dang Quang, Doan Thanh Phuong, Pham Thu Hang, Tran Huu Loc, Nguyen Thi Ngoc Lan, Nguyen Huy Dung, Jeremy Farrar, Maxine Caws

**Affiliations:** Pham Ngoc Thach Hospital, Ho Chi Minh City, Vietnam; Oxford University Clinical Research Unit, Ho Chi Minh City, Vietnam; Department of Clinical Sciences, Liverpool School of tropical Medicine, Pembroke Place, L3 5QA UK

**Keywords:** Tuberculosis, Pediatric, Childhood, Xpert, Genexpert, Diagnosis, MGIT culture, Smear

## Abstract

**Background:**

The GeneXpertMTB/RIF (Xpert) assay is now recommended by WHO for diagnosis of tuberculosis (TB) in children but evaluation data is limited.

**Methods:**

One hundred and fifty consecutive HIV negative children (<15 years of age) presenting with suspected TB were enrolled at a TB referral hospital in Ho Chi Minh City, Vietnam. 302 samples including sputum (n = 79), gastric fluid (n = 215), CSF (n = 3), pleural fluid (n = 4) and cervical lymphadenopathic pus (n = 1) were tested by smear, automated liquid culture (Bactec MGIT) and Xpert.

Patients were classified retrospectively using the standardised case definition into confirmed, probable, possible, TB unlikely or not TB categories. Test accuracy was evaluated against 2 gold standards: [1] clinical (confirmed, probable and possible TB) and [2] ‘confirmed TB’ alone.

**Results:**

The median age of participants was 18 months [IQR 5–170]. When test results were aggregated by patient, the sensitivity of smear, Xpert and MGIT against clinical diagnosis as the gold standard were 9.2% (n = 12/131) [95%CI 4.2; 14.1], 20.6% (n = 27/131) [95%CI 13.7; 27.5] and 29.0% (n = 38/131) [21.2;36.8], respectively. Specificity 100% (n = 19/19), 94.7% (n = 18/19), 94.7% (n = 18/19), respectively. Xpert was more sensitive than smear (P = <0.001) and less sensitive than MGIT (P = 0.002).

**Conclusions:**

The systematic use of Xpert will increase early TB case confirmation in children and represents a major advance but sensitivity of all tests remains unacceptably low. Improved rapid diagnostic tests and algorithm approaches for pediatric TB are still an urgent research priority.

## Background

Pediatric tuberculosis (TB) is a neglected disease. There were an estimated 9 million new cases and 1.5 million deaths each year from tuberculosis worldwide in 2013 [1]. The case burden in children is extremely difficult to estimate due to the difficulty in confirming a diagnosis and consequent lack of notification data through most National TB Programmes. In the last five years there has been a co-ordinated effort by the research community to address the lack of research on pediatric TB, including evaluation of new diagnostics, development of pediatric drug formulations and inclusion of children in clinical intervention trials [2]. The most recent estimates from WHO are over half a million TB cases and 74,000 deaths among children without HIV infection each year [3].

Diagnosis of childhood TB is difficult and microbiological confirmation by smear is rare. Children typically are unable to expectorate sputum or produce small quantities. Few bacilli are present in the respiratory secretions and sputum smear has a limit of detection of approximately 5,000-10,000 acid fast bacilli (AFB)/ml [4]. In addition, recognition of TB disease in children is complicated by the fact that clinical signs and symptoms are less specific than in adult disease [5-7].

The best available diagnostic tests are costly, while traditional methods are slow or insensitive. In facilities with access to the full range of diagnostic tools, *Mycobacterium tuberculosis* (*M. tuberculosis*) is isolated from fewer than half of children ultimately treated for TB [8-11]. Scoring systems and algorithm approaches have been proposed but in the absence of microbiological confirmation the decision to treat ultimately rests on clinical experience in conjunction with tools available since the 1940s: tuberculin skin test (TST), and chest X-ray (CXR), in addition to history and physical exam [12,13].

The GeneXpertMTB/RIF (Cepheid, USA) assay is a nucleic acid amplification (NAAT) test that can simultaneously identify *M. tuberculosis* complex bacteria and resistance to rifampicin (RIF). The test was endorsed by WHO for the diagnosis of TB in 2011 but due to limited evaluation data there was no specific recommendation for its use in pediatric cases [14]. In October 2013, an updated systematic review resulted in the recommendation that Xpert should be used rather than conventional microscopy as the initial diagnostic test in children suspected of having MDR TB or HIV associated TB (strong recommendation) and that Xpert may be used rather than conventional microscopy and culture as the initial test in all children suspected of having TB (conditional recommendation acknowledging resource limitations, very low quality of evidence) [15]. Much of the data on Xpert for diagnosis of TB in children has come from South Africa and there remains a need for further evaluations in diverse settings. Therefore, we undertook a prospective study to evaluate Xpert for the diagnosis of TB in HIV uninfected children at a tertiary referral TB hospital in Vietnam. Xpert was compared with homogenous sputum smear and commercial liquid culture using the standardised case definition [16].

## Methods

Pham Ngoc Thach hospital (PNT) is a 900 bed tertiary referral hospital for TB and Lung Diseases in Ho Chi Minh City, Vietnam. There is a 70 bed pediatric ward within the hospital which treats the local community and also receives referrals from throughout the 21 provinces of southern Vietnam, including the two large pediatric hospitals in the city: Nhi Dong 1 and Nhi Dong 2.

Enrollment*:* Any child (≤15 years of age) presenting at the pediatric ward of Pham Ngoc Thach hospital, Ho Chi Minh City, with suspected pediatric TB was eligible to join the study if they were HIV negative and had not been given TB drugs in the current illness episode prior to recruitment. Consecutive patients to a target sample size of 150 were recruited. An average of 2 samples per child was anticipated based upon a previous study in the same setting, which would yield 300 samples from 150 children. Assuming a sensitivity of 30% for smear and 45% for GeneXpert, 230 samples would be required to detect a difference in sensitivity with 90% power, alpha = 0.05.

Routine diagnostic samples were collected as judged appropriate by the treating clinician and all sample types were eligible for inclusion in the study including gastric aspirate (GA)/broncho-alveolar lavage (BAL), sputum, cerebral spinal fluid (CSF), nasophagyngeal aspirate (NPA), pleural fluid. No additional samples were collected from the patients for the purposes of this study.

In children suspected of TB meningitis (TBM), it was recommended that the largest volume of CSF which could safely be collected, as judged by the treating clinician, was drawn for mycobacterial testing.

CXRs (2 views) were interpreted by 2 independent pediatric radiologists who are experienced in reviewing CXRs in children. In the case of discordant reading, a third expert reader reviewed the CXR and a final consensus achieved.

HIV testing was performed as part of routine care for suspected pediatric TB cases.

The TST using the Mantoux method was performed according to standard protocols [17]. Five tuberculin units (TU) of tuberculin PPD-S were used for the TST. The results were read 72–96 hours after injection. The diameter of indurations (thickening of the skin) in millimeters was recorded. >5 mm was considered positive.

All specimens were collected before starting anti-TB therapy.

Clinical case definition categories for TB in children were determined retrospectively and taken from the standardised case definition recently published by Graham et al. [16] as follows:

*‘Confirmed TB cases’* were defined as children with at least 1 defined sign or symptom suggestive of TB and microbiologically confirmed TB, defined as at least one positive smear or MGIT in any sample. A positive Xpert was not considered as part of the ‘Confirmed TB’ case defintion because this was the research test under evaluation.

*‘Probable TB cases’* were defined as children with at least 1 defined sign or symptom suggestive of TB and a CXR consistent with TB and at least 1 of the following: [1] positive clinical response to TB therapy [3] documented exposure to a household or close contact with a TB case or [18] positive TST.

*‘Possible TB cases’* were defined as children with at least 1 sign or symptom suggestive of TB and who had either: [1] a CXR that is not consistent with TB and at least 1 of the following: positive clinical response to TB therapy, documented exposure to a household or close contact with a TB case or positive TST or [3] a CXR consistent with TB but none of the other characteristics listed in [1].

‘TB unlikely’ cases were those who are symptomatic with symptoms other than the defined TB symptoms and who do not fit the above definitions with no alternative diagnosis confirmed.

‘Not TB’ cases were defined as those who fitted the diagnosis for ‘TB unlikely’ and also had an alternative diagnosis established (microbiologically or recovery without antituberculous therapy).

The ‘TB unlikely’ and ‘Not TB’ groups were combined as negative under the clinical TB gold standard for analysis.

TB signs and symptoms are defined as persistent unexplained fever, persistent cough (>2 weeks), night sweats, weight loss, failure to thrive, reduced playfulness or lethargy, neonatal pneumonia, unexplained hepatosplenomegaly or sepsis like illness. For full definitions of symptoms see reference [16].

Definitions of TB treatment outcomes were according to standard World Health Organization (WHO) definitions [18,19]: Cured, treatment completed, default, transfer out or died.

### Sample processing

All samples, except CSF, were decontaminated by Sputaprep (NaOH –NALC 2%, Nam Khoa Company-Viet Nam) before testing. Briefly, an equal volume of NaOH-NALC was added to the sample tube and vortexed for 20 minutes. Sterile water was then added to reach a final volume of 45 ml. The tube was then centrifuged at 3000 g for 15 minutes, the supernatant discarded and the pellet used for testing. CSF was not decontaminated before centrifugation. All sample pellets (including CSF pellet) were then divided for smear microscopy, MGIT culture and Xpert assay. Technicians interpreting the Xpert assay were blind to clinical data and to other test results.

#### Ziehl-Neelsen (ZN) smear

Two drops of sample pellet (approximately 200 μl) were used for smear microscopy (ZN staining), according to the WHO standard protocol [20].

#### MGIT

Five hundred microliters of each deposit were inoculated into a MGIT tube, following the manufacturer’s protocol, and incubated in a Bactec MGIT 960 system at 37°C. Results were automatically reported by the system. Positive cultures were tested by ZN smear to confirm the presence of acid fast bacilli. BD MGIT™ TBc Identification Test which detects MPT64 antigen (Becton Dickinson, USA) was performed for TB identification.

#### Xpert MTB/RIF

0.5 ml of each sample deposit was treated with 1.5 ml of sample reagent and processed according to manufacturer’s standard operating procedure (SOP) (Cepheid, USA).

DST testing: The first positive MGIT culture for each patient was tested by indirect phenotypic drug susceptibility testing (DST) for the first line TB drugs by 1% proportional method on Lowenstein Jensen media. DST was performed for isoniazid (0.2 μg/ml), streptomycin (4 μg/ml), rifampicin (40 μg/ml), ethambutol (2 μg/ml) and pyrazinamide (Wayne method, 200 μg/ml), at the TB reference laboratory at PNT, which is accredited by the WHO TB reference laboratory of Western Pacific region (Adelaide, Australia).

### Ethics

Eligible children were invited to participate in the study through their parents who gave written informed consent following consultation. The protocol, parental informed consent form (ICF) and case report form (CRF) were approved by the PNT hospital Institutional Review Board (IRB), the Oxford Tropical Ethics Committee (OxTREC) and the Health Services of Ho Chi Minh City.

### Statistical analysis

Accuracy measures (sensitivity, specificity, positive and negative predictive values) of the 3 tests were calculated for 2 different definitions of gold standard: [1] ‘confirmed TB’ gold standard and [3] ‘clinical gold standard’ (including confirmed*,* probable and possible *TB cases*). The ‘TB unlikely’ and ‘Not TB’ groups were combined as clinically negative for analysis. Two gold standards were applied for the analysis as it is known that microbiological confirmation detects only approximately half of all pediatric TB cases when applied optimally and will therefore overestimate sensitivity and underestimate specificity. Conversely, a perfect clinical gold standard does not exist and therefore clinical gold standards are likely to underestimate sensitivity while overestimating specificity. This is a well-recognised problem in the evaluation of novel diagnostic tests for TB and particularly acute for pediatric TB and other paucibacillary manifestations. The use of standardised clinical definitions aims to improve comparibility between reports on the evaluation of diagnostic tests and facilitate meta-analysis, but all TB algorithm case definitions have limitations and should not be considered to define cases for treatment.

In addition, the data were analyzed both on the ‘per patient’ and the ‘per sample’ level. For the ‘per patient’ analysis, all samples of the patient were aggregated to a single test result which was defined as ‘positive’ if the test was positive for at least one of the samples. To account for potential correlation between multiple samples per patient and different tests within the same sample or patient, marginal binomial regression models with an identity link function and associated robust standard error estimates were used to estimate accuracy measures of smear, MGIT, and Xpert and corresponding 95% confidence intervals (95%CI), as well as to compare the accuracy measures between these tests.

Demographic and clinical characteristics of patients were compared between diagnosed categories of TB (confirmed, probable, possible) and clinically negative (‘TB unlikely’ and ‘not TB’ combined). Fisher’s exact test (for categorical variables) and Kruskal Wallis test (for continuous variables) were used for both overall and pairwise comparisons between groups.

All analyses were done with R version 3.1.0 (R Foundation for statistical computing, Vienna, Austria). Two-sided p-values <0.05 were regarded as statistically significant.

## Results

**Figure 1:**
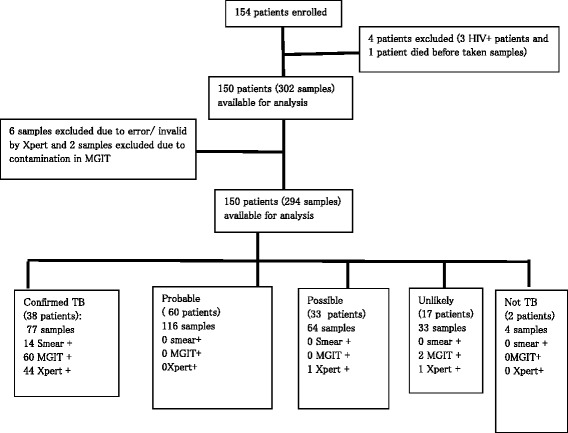
**Flow-chart of patient enrolment and analysis.**

From April to October 2013, a total of 154 suspected childhood TB cases were enrolled into the study. Four children were excluded from the study; (3 children infected with HIV detected after enrolment and 1 child who died before diagnostic samples were obtained). Data of 150 children were available for analysis. A recruitment flow chart is shown in Figure 1.

In total, 302 samples were collected: sputum (n = 79), gastric fluid (n = 215), CSF (n = 3), pleural fluid (n = 4) and cervical lymphadenopathic pus (n = 1). The treating clinician determined the number and type of samples collected. For children aged five years or younger (0–59 months of age), the most common sample type was gastric aspirate (209/221, 94.6%), while for older children (60–179 months of age), sputum was normally collected (72/81, 88.9%). Only ten children had two different sample types.

Among 302 samples from 150 children tested, there were 6/302 (2.0%) Xpert tests with invalid reports and 2/302 (0.7%) contaminated MGIT tests. These samples were excluded from further analysis, resulting in a total of 294 samples but this did not decrease the number of patients included (n = 150).

Thirty eight patients (25.3%, n = 38/150) were classified as ‘confirmed TB’ , 60 patients (40.0%, n = 60/150) were ‘probable TB’ cases, 33 (22.0% n = 33/150) patients were ‘possible TB’ cases, 17 patients were ‘TB unlikely’ (11.3%, n = 17/150) and 2 patients (1.3%, n = 2/150) were classified as ‘Not TB’ with an alternative confirmed diagnosis.

General demographic characteristics of the study population are shown in Table 1. Overall, the median age of children in the study population was 18.5 months. Boys were marginally younger (median = 18 [IQR 9–45.75]) than girls (median = 21.5 [IQR 11.75-128.5]). Over two-thirds of these children (n = 109/150, 72.7%) were between 0 and 4 years old. The boy: girl ratio was approximately 2:1 (n = 98/52), consistent with the gender inequality seen in adult TB.

**Table 1:** **Demographic characteristics of patients**

	**Total**	**Confirmed TB**	**Probable TB**	**Possible TB**	**TB unlikely**	**Not TB**
	**N = 150 (%)**	**N =38 (%)**	**N = 60 (%)**	**N = 33 (%)**	**N = 17 (%)**	**N = 2 (%)**
Gender	P* = 0.3237					
Boy	98 (65.3)	23 (60.5)	40 (66.7)	25 (75.8)	10 (52.6)	1 (50.0)
Girl	52 (34.7)	15 (39.5)	20 (33.3)	8 (24.2)	9 (41.4)	1 (50.0)
Age (months) Median [IQR]	18.5[5–170]	121.5[4.9-173]	14[4.95-159]	18[5–160.4]	19[7.6-171]	41[32–50]
Weight (kg) Median [IQR]	10[5–34.4]	24.5[6.4-47.4]	8.7[5.3-37.1]	10[5.6-39]	10.2[6.8-46.1]	11.9[6.4-47.5]
Height (cm) Median [IQR]	83[60–162]	131[63–162.8]	78[61–157.4]	78[59.7-160]	83 [67–155.8]	91[84.7-97.3]
BCG	P* = 0.6129					
Yes	133 (88.7)	29 (76.3)	55 (91.7)	31 (93.9)	18 (94.7)	2 (100)
No	16 (10.7)	8 (21.1)	5 (8.3)	2 (6.1)	1 (5.3)	0 (0)
Unknown	1 (0.67)	1 (2.63)	0 (0)	0 (0)	0 (0)	0 (0)
TB contact	P* < 0.001					
Yes	30 (20.0)	12 (31.6)	16 (26.7)	2 (6)	0 (0)	0 (0)
Family member	27 (90.0)	11 (91.7)	14 (87.5)	2 (100)	0 (0)	0 (0)
No	120 (80.0)	26 (68.4)	42 (73.3)	31 (94.0)	19 (100)	2 (100)

*P value for comparison of all four groups.

Evidence of BCG vaccination was recorded in 89% (n = 133/150) (scar or parent report). Neonatal BCG vaccination is compulsory under the Expanded Vaccination Program (EVP) of Vietnam. One-fifth (n = 30/150) of the study population had a TB contact according to parent interview and of those contacts 90% (n = 27/30) were a household member. The confirmed TB patients reported TB close contact more often (P = 0.01) than the clinically negative group (‘TB unlikely’ + ‘not TB’ patients combined).

The clinical manifestations of TB reported in this referral study population included fever (82.7%), persistent cough (79.3%), night sweats (68.0%), weight loss (42.7%), failure to thrive (40.7%), reduced playfulness (29.3%), lymphadenopathy (21%) and CXR consistent with TB (95.3%) (Table 2). There were no significant differences by clinical groups in the TB signs and symptoms recorded (P-value >0.05). Other symptoms were haemoptysis (4%), wheezing (2.67%), difficulty breathing (1.3%) and vomiting. With the exception of a single child who may have had an unconfirmed history of TB, all of the children were new TB patients. Over 70% (n = 111/150) of the children had a TST; 30/111 (27.0%) were positive. The median history of illness for all children in the study was 30 days [IQR 6.45-90].

**Table 2:** **Clinical characteristics of 150 patients by final clinical classification**

	**Total**	**Confirmed TB**	**Probable TB**	**Possible TB**	**TB unlikely**	**Not TB**
	**N = 150 (%)**	**N = 38 (%)**	**N = 60 (%)**	**N = 33 (%)**	**N = 17 (%)**	**N = 2 (%)**
History of illness (days) Median [IQR]	30[6.45-90]	30[6.4-94.5]	30[8–90]	17[6.6-72]	30[2–60]	30[30–30]
Fever (>38°C)	P = 0.038					
≥1 week	82 (54.7)	22 (58.0)	39 (65.0)	15 (45.5)	5 (29.4)	1 (50)
<1 week	42 (28)	8 (21.0)	14 (23.3)	14 (42.4)	6 (35.3)	0 (0)
No fever	26 (17.33)	8 (21.0)	7 (11.7)	4 (12.1)	6 (35.3)	1(50)
Persistent cough	P = 0.340					
Yes	119 (79.3)	30 (79.0)	53 (88.3)	23 (70.0)	11 (64.7)	2 (100)
No	31 (20.7)	8 (21.0)	7 (11.7)	10 (30.0)	6 (35.3)	0 (0)
Night sweats	P = 0.825					
Yes	102 (68.0)	21 (55.3)	44 (73.3)	25 (75.8)	10 (58.8)	2 (100)
No	48 (32.0)	17 (44.7)	16 (26.7)	8 (24.2)	7 (41.2)	0 (0)
Weight loss	P = 0.763					
Yes	64 (42.7)	17 (44.7)	28 (46.7)	12 (36.4)	6 (35.5)	2 (100)
No	86 (57.3)	21 (55.3)	32 (53.3)	21 (63.6)	11 (64.7)	0 (0)
Failure to thrive	P =0.378					
Yes	61 (40.7)	8 (21.0)	30 (50.0)	13 (39.4)	8 (47.1)	2 (100)
No	89 (59.3)	30 (79.0)	30 (50.0)	20 (60.6)	9 (52.9)	0 (0)
Reduced playfulness	P = 0.969					
Yes	44 (29.3)	9 (23.7)	18 (30.0)	12 (36.4)	4 (23.5)	1 (50)
No	106 (70.7)	29 (76.3)	42 (70.0)	21 (63.6)	13 (76.5)	1 (50)
Lymphadenopathy	P = 0.910					
Yes	21 (14.0)	9 (23.7)	6 (10.0)	4 (12.1)	1 (5.9)	1 (50)
No	129 (86.0)	29 (76.3)	54 (90.0)	29 (87.9)	16 (94.1)	1 (50)
Chest X ray	P = 0.060					
Consistent with TB	143 (95.3)	37 (97.4)	60 (100)	30 (90.9)	14 (82.4)	2 (100)
Not consistent with TB	5 (3.33)	0 (0)	0 (0)	3 (9.1)	2 (11.8)	0
Unclear	2 (1.3)	1 (2.6)	0 (0)	0 (0)	1 (5.2)	0
TST	P = 0.3171					
<5 mm	81 (73.0)	15 (60.0)	32 (66.7)	29 (87.9)	5 (100)	0
≥5 mm - < 10 mm	17 (15.3)	5 (20.0)	8 (16.7)	4 (12.1)	0	0
≥10 mm	13 (11.7)	5 (20)	8 (16.7)	0	0	0
Not done	39 (26.0)	13 (34.2)	12 (20.0)	0	12 (70.6)	2 (100)

### Accuracy of Xpert

#### Clinically diagnosed TB as the gold standard

The clinically diagnosed gold standard was defined as all patients in the ‘confirmed TB’ , ‘probable TB’ and ‘possible TB’ groups combined. One hundred and thirty one patients satisfied the criteria for clinically diagnosed TB and 19 patients were clinically negative (classified as TB unlikely (n = 17) or not TB (n = 2)) (Figure 1).

### By patient analysis

When analyzed by patient against the clinical gold standard, the sensitivity of smear, MGIT and Xpert were 9.2% [95%CI: 4.2, 14.1] (n = 12/131); 29.0% [95%CI: 21.2, 36.8] (n = 38/131) and 20.6% [95%CI: 13.7, 27.5] (n = 27/131), respectively. Xpert was more sensitive than smear (P = <0.001) [95%CI of difference: −5.6%; −17.3%] and less sensitive than MGIT (P = 0.002 [95%CI of difference: 3.2%; 13.6%].

Specificity and positive predictive value (PPV) of smear were 100% (n = 19/19 and n = 12/12). Specificity and PPV of MGIT were 94.7% [95%CI: 84.7; 100] (n = 18/19) and 97.4% [95%CI: 92.4, 100] (n = 38/39).

Specificity and PPV of Xpert were 94.7% [95%CI: 84.7; 100] (n = 18/19) and 96.4% [95%CI: 89.6, 1.03] (n = 27/28), respectively.

The negative predictive value (NPV) of smear, MGIT, Xpert were 13.7% [95%CI: 8.0, 19.5] (n = 19/138); 16.2% [95%CI: 9.4, 23.1] (n = 18/111) and 14.8% [95%CI: 8.5, 21.1] (n = 18/122), respectively.

Relative to smear, 15 additional cases were detected by Xpert, while MGIT detected 26 additional cases over smear. There were 11 cases detected by MGIT which were not detected by Xpert. Conversely, a single case of possible TB was detected by Xpert which was not detected by MGIT. There was also a single patient in the ‘TB unlikely’ group positive by both MGIT and Xpert. This patient did not have any standard signs/symptoms of TB and therefore did not meet the case definition for confirmed/probable/possible TB despite having a positive MGIT culture.

Accuracy of smear, MGIT and Xpert by patient were 20.6% (31/150), 37.3% (56/150) and 30.0% (45/150), respectively.

### By sample analysis

When analyzed by sample, the sensitivity of smear, MGIT and Xpert were 5.4% [95%CI: 2.2, 8.7] (n = 14/257); 23.3% [95%CI: 16.7, 30.0] (n = 60/257) and 17.5% [95%CI: 11.3, 23.8] (n = 45/257), respectively.

The sensitivity of Xpert was significantly higher than smear (P = <0.001) [95%CI of difference: −16.6%.9%, −6.9%] and significantly lower than MGIT (P = <0.001) [95%CI of difference: 2.8%; 9.6%]. Table 3 shows the sensitivity of the three tests against clinical diagnosis as gold standard in terms of patients, samples and type of sample.

**Table 3:** **The sensitivity, specificity, positive predictive value (PPV) and negative predictive value (NPV) of smear, MGIT and Xpert for the diagnosis of pediatric TB**

		**Sensitivity% (x/n); [95%CI]**	**Specificity% (x/n); [95%CI]**	**PPV% (x/n); [95%CI]**	**NPV% (x/n); [95%CI]**
Gold standard 1 = Confirmed TB	**Per patient analysis (n = 150)**				
	Xpert	68.4 (26/38) [53.6, 83.2]	98.2 (110/112) [95.7; 100]	92.9 (26/28) [83.3; 100]	90.2 (110/122) [84.9, 95.5]
	Smear	31.6 (12/38) [16.8; 46.4]	100 (112/112)*	100 (12/12)*	81.2 (112/138) [74.6; 87.7]
	**Per sample analysis (n = 294)**				
	Xpert	57.1 (44/77) [42.8, 71.5]	99.1 (215/217) [97.8, 1.00]	95.7 (44/46) [89.7, 100]	86.8 (219/248) [81.0; 92.6]
	Smear	18.2 (14/77) [8.5; 27.9]	100 (215/217)	100 (14/14)	77.5 (217/280) [70.7; 84.3]
Gold standard 2 = Clinical diagnosis	**Per patient analysis (n = 150)**				
	Smear	9.2 (12/131) [4.2, 14.1]	100 (19/19)	100 (12/12)	13.8 (19/138) [8.0, 19.5]
	MGIT	29.0 (38/131) [21.2, 36.8]	94.7 (18/19) [84.7, 100]	97.4 (38/39) [92.5, 100]	16.2 (18/111) [9.4, 23.1]
	Xpert	20.6 (27/131) [13.7, 27.5]	94.7 (18/19) [84.7,100]	96.4 (27/28) [89.6,100]	14.8 (18/122) [8.5, 21.1]
	**Per sample analysis (n = 294)**				
	Smear	5.4 (14/257) [2.2; 8.7]	100 (37/37)	100 (14/14)	13.2 (37/280) [7.6; 18.8]
	MGIT	23.3 (60/257) [16.7; 30.0]	94.6 (35/37) [84.3; 100]	96.8 (60/62) [90.6; 100]	15.1 (35/232) [8.6; 21.5]
	Xpert	17.5 (45/257) [11.3; 23.8]	97.3 (36/37) [92.1; 100]	97.8 (45/46) [93.6; 100]	14.5 (36/248) [8.4; 20.6]

*95%CI could not be calculated based on marginal logistic regression.

Specificity of smear, MGIT and Xpert were 100% (n = 37/37); 94.6% [95%CI: 84.3, 100] (n = 35/37) and 97.3% [95%CI: 92.1, 100] (n = 36/37), respectively. The PPV of smear, MGIT and Xpert were 100% (n = 14/14); 96.8% [95%CI: 90.6; 100] (n = 60/62) and 97.8% [95%CI: 93.6; 1.02] (n = 45/46), respectively. The NPV of smear, MGIT and Xpert were 13.2% [95%CI: 7.6; 18.8] (n = 37/280); 14.8% [95%CI: 8.6; 21.5] (n = 35/232) and 14.5% [95%CI: 8.4; 20.6] (n = 36/248), respectively.

Accuracy of smear, MGIT and Xpert by sample were 19.8% (51/257), 37.0% (95/257) and 31.5% (81/257), respectively.

### Confirmed TB as the gold standard

#### Per patient analysis

Of 38 patients classified in the ‘confirmed TB’ group, 26 patients (68.4%) had at least one sample positive by Xpert.

When analyzed by patient, the sensitivity, specificity, PPV and NPV of Xpert were 68.4% [95%CI: 53.6, 83.2] (n = 26/38); 98.2% [95%CI: 95.7; 100] (n = 110/112); 92.9% [95%CI: 83.3; 100] (n = 26/28) and 90.2% [95%CI: 84.9; 95.5] (n = 110/122), respectively against ‘confirmed TB’ as the gold standard.

### Per sample analysis

With analysis by sample, Xpert had a sensitivity of 57.1% [95%CI: 42.8; 71.5] (n = 44/77), a specificity of 99.1% [95%CI: 97.8, 100] (n = 215/217), a PPV of 95.7% [95%CI: 89.7; 100] (n = 44/46) and a NPV of 86.8% [95%CI: 81.0; 92.6] (n = 219/248).

Table 3 summarizes the sensitivity, specificity, PPV and NPV of three tests for the diagnosis of pediatric TB in terms of ‘confirmed TB’ and clinical gold standards.

### Sensitivity by sample type

For sputum (n = 78), the sensitivity of Xpert (41.7%, n = 30/72) was significantly higher than smear (12.5%, n = 9/72), P <0.001 [95%CI of difference: −24.6%; −16.5%] and lower than MGIT (50.0%, n = 36/72), P = 0.002 [95%CI of difference: 1.0%; 1.6%].

For gastric fluid (n = 209), the sensitivity of Xpert (7.7%, n = 14/181) was more than three times that of smear (2.2%, n = 4/181) [P = <0.005 95% CI of difference: −8.5%; −1.5%] but lower than MGIT (13.3%, n = 24/181).[P = <0.005; 95%CI of difference: 1.6%; 8.5%].

There were insufficient numbers of other sample types for a robust analysis: pleural fluid (n = 3), CSF (n = 3) and cervical lymphadenopathic pus (n = 1).

The number of Xpert positive results by both patient and sample across the spectrum of diagnostic certaintity is shown in Table 4 [21].

**Table 4:** **Xpert results by certainty of diagnosis**

		**Confirmed TB n (%)**	**Probable TB n (%)**	**Possible TB n (%)**	**TB unlikely n (%)**	**Not TB n (%)**	**Total N (%)**
**By patient**							
	**Xpert positive**	**26 (68.4)**	**0 (0)**	**1 (3.0)**	**1 (5.9)**	**0 (0)**	**28 (18.7)**
	**Xpert negative**	**12 (31.6)**	**60 (100)**	**32 (97.0)**	**16 (94.1)**	**2 (100)**	**122 (81.3)**
	**Total**	**38 (100)**	**60 (100)**	**33 (100)**	**17 (100)**	**2 (100)**	**150 (100)**
**By Sample**							
	**Xpert positive**	**44 (57.1)**	**0 (0)**	**1 (1.6)**	**1 (3.0)**	**0 (0)**	**46 (15.6)**
	**Xpert negative**	**33 (42.9)**	**116 (100)**	**63 (98.4)**	**32 (97.0)**	**4 (100)**	**248 (84.3)**
	**Total**	**77 (100)**	**116 (100)**	**64 (100)**	**33 (100)**	**4 (100)**	**294 (100)**

### Resistance to first line drugs

Two samples from 2 different patients were positive for RIF resistant strains by Xpert testing.

The first patient was a 6 month old girl with a 15 day history of persistent cough, night sweats and vomiting after breastfeeding. No TB contact was recalled by the parents. A CXR was obtained, which demonstrated an infiltrate consistent with TB near the right-side lung hilar. Phenotypic DST on the isolate from gastric fluid culture in MGIT culture showed resistance to streptomycin and rifampicin but susceptibility to isoniazid, ethambutol and pyrazinamide.

The second patient was a 12 year old girl presenting to PNT with 1 month of persistent cough, fever > 38°C and weight loss. She had been living in the same house with an adult pulmonary TB case. The CXR showed a lesion consistent with TB at right-side lung apex. Sputum was smear negative, but positive in both MGIT and Xpert assays. Phenotypic DST showed susceptiblity to all first line drugs. The phenotypic DST result was taken as gold standard by the treating clinician and the patient was treated with 2RHZE/4RH. Seven months after treatment completion, the patient is thriving and has not relapsed.

Fifty five samples from 32 patients were sent for phenotypic DST. One sample from each patient was chosen to perform DST, if this sample was contaminated, the second sample of the same patient was tested.

Three out of 32 patients (9.4%) had a contaminated MGIT culture, 19/32 (59.4%) patients had an isolate susceptible to all four drugs and 10/29 (34.5%) patients had an isolate resistant to at least one drug including 6 cases (60.0%, n = 6/10) which were polyresistant (4 cases resistant to streptomycin and isoniazid, 1 case resistant to streptomycin and rifampicin and another one resistant to isoniazid and ethambutol) and 4 cases (40.0%, n = 4/10) were monoresistant to streptomycin).

There were no cases of RIF resistant TB on phenotypic testing which were not detected by Xpert.

## Discussion

This study confirms that Xpert is a suitable, rapid and specific method for the diagnosis of childhood TB with approximately twice the sensitivity of smear microscopy.

With clinical diagnosis as the gold standard, Xpert detected 20.6% of children with clinically diagnosed TB, an 11% increase over smear (P = <0.001). MGIT culture detected substantially more cases than Xpert (38 vs 26, respectively) (P = 0.002) but is not a rapid test.

The high proportion of children in the study who were eventually diagnosed with TB reflects the setting of a tertiary referral hospital for TB and the proportion of TB cases in a general hospital would be far lower. It is important to evaluate novel diagnostic tests in multiple settings: at referral and general hospitals as well as at clinic level, particularly for diseases which are relatively rare at a population level, as is pediatric TB. The performance characteristics of a test may be affected by numerous factors including the differential diagnostic spectrum, pre-selection criteria, the disease prevelence in the tested population, sample processing and experience of technicians performing the test. Ideally, a diagnostic test performance will be robust to these characteristics in routine use. The sensitivity of Xpert for the detection of pediatric TB in this study is consistent with two previous studies from South Africa (20.3%) and Tanzania (33.3%) [22-24]. All of these studies show that Xpert substantially increases detection of pediatric TB over the smear technique. Although MGIT culture remains the most sensitive technique and provides valuable confirmation of diagnosis, the results are too slow to aid in acute treatment decisions. The limit of detection of the Xpert assay is 131 colony forming units (cfu)/ml [14] and it is likely that MGIT culture is able to detect positive samples which are below this limit. Alternative sample processing strategies which may enrich the DNA extraction of Xpert should be evaluated.

Childhood TB samples are often paucibacillary and therefore it is very important to establish which sample types or induction methods yield the highest sensitivity. Although the sensitivity of Xpert was higher in sputum than gastric fluid in this study, this was likely due to the older age of children able to produce sputum and a direct comparison cannot be made. Results from studies using systematic multiple sampling strategies, including the string test, induced sputum, nasopharyngeal aspirate and stools, which are now ongoing will yield important insights into the optimal sampling strategies for Xpert testing for children [25-28].

The use of a standardised case definition should facilitate comparisons between studies and is an important advance in the field of pediatric TB research. However, there was a single case in this study which was both MGIT culture and Xpert positive from pleural fluid but did not exhibit any of the defined ‘signs suggestive of TB’ and was therefore classified as ‘TB unlikely’ rather than confirmed by application of the standardised case defintion. This case was tested for TB due to dyspnoea and pleural effusion on chest X-ray and was treated for TB by the ward clinician with improvement on treatment. While it remains a possibility that both Xpert and MGIT cultures were false positive due to contamination at sampling, this is a recognised presentation in older children and this case highlights the diversity of presentation in childhood TB and the difficulty of using standardised definitions to classify cases. The standardised case definition is currently being revised to improve classification, however it will always be difficult to emcompass the broad spectrum of possible presentation for pediatric TB. Ultimately clinical judgment must be used to determine treatment in unusual cases. If this case had been classified as ‘confirmed TB’, the sensitivity of Xpert would have increased only marginally (21.2% vs 20.6%). We were not able to apply minimum follow up times as the study did not prescribe changes to normal routine practice and patients were often routinely discharged prior to the recommended two week follow-up time. This may have resulted in the misclassification of some cases.

Test failure (invalid and error reports) has important cost implications. The Xpert failure rate was acceptably low (2%, n = 6/302) in this study and comparable with reports from demonstration sites [29].

In the absence of bacteriologic confirmation, the diagnosis of TB in children still rests on the triad of (i) thorough history, especially a history of contact with a known TB case, (ii) a positive TST and (iii) signs suggestive of TB on CXR. Although MDR TB in children remains relatively rare, the consequences of delayed diagnosis are grave. The ability to rapidly detect RIF resistance in this vulnerable population is a major advance, but care must be taken, in light of the low positive predictive value in populations with a low MDR prevalence [30,31] to obtain confirmatory testing. The small number (n = 2) of RIF resistant cases detected in this study does not allow robust conclusions to be drawn. The false positive RIF resistant result in this study does however highlight the risk of false positive MDR diagnosis. Both the consequences of inappropriate MDR treatment initiation and delayed treatment of true MDR are likely to have more severe complications in childhood cases. Any RIF resistant result should be interpreted in the light of risk factors and confirmed by a second test.

## Conclusion

The Xpert assay increases rapid confirmation of pediatric TB substantially and should be applied to this vulnerable population. Despite this, over 50% of clinically treated cases are unconfirmed and further research on the diagnosis and treatment of childhood TB should remain a priority of the global health community.
